# Gene fragmentation in bacterial draft genomes: extent, consequences and mitigation

**DOI:** 10.1186/1471-2164-13-14

**Published:** 2012-01-10

**Authors:** Jonathan L Klassen, Cameron R Currie

**Affiliations:** 1 Department of Bacteriology, University of Wisconsin--Madison, Madison, Wisconsin, USA; 2 DOE Great Lakes Bioenergy Research Center, University of Wisconsin--Madison, Madison, Wisconsin, USA

## Abstract

**Background**

Ongoing technological advances in genome sequencing are allowing bacterial genomes to be sequenced at ever-lower cost. However, nearly all of these new techniques concomitantly decrease genome quality, primarily due to the inability of their relatively short read lengths to bridge certain genomic regions, e.g., those containing repeats. Fragmentation of predicted open reading frames (ORFs) is one possible consequence of this decreased quality. In this study we quantify ORF fragmentation in draft microbial genomes and its effect on annotation efficacy, and we propose a solution to ameliorate this problem.

**Results**

A survey of draft-quality genomes in GenBank revealed that fragmented ORFs comprised > 80% of the predicted ORFs in some genomes, and that increased fragmentation correlated with decreased genome assembly quality. In a more thorough analysis of 25 *Streptomyces *genomes, fragmentation was especially enriched in some protein classes with repeating, multi-modular structures such as polyketide synthases, non-ribosomal peptide synthetases and serine/threonine kinases. Overall, increased genome fragmentation correlated with increased false-negative Pfam and COG annotation rates and increased false-positive KEGG annotation rates. The false-positive KEGG annotation rate could be ameliorated by linking fragmented ORFs using their orthologs in related genomes. Whereas this strategy successfully linked up to 46% of the total ORF fragments in some genomes, its sensitivity appeared to depend heavily on the depth of sampling of a particular taxon's variable genome.

**Conclusions**

Draft microbial genomes contain many ORF fragments. Where these correspond to the same gene they have particular potential to confound comparative gene content analyses. Given our findings, and the rapid increase in the number of microbial draft quality genomes, we suggest that accounting for gene fragmentation and its associated biases is important when designing comparative genomic projects.

## Background

Beginning with the bacteriophage φX174 genome in 1977 by Sanger et al. [1], microbes have spearheaded technological advances in genome sequencing. This is almost certainly because their small size and limited genomic complexity (at least compared to Eukaryotes) makes genomic analysis especially tractable, and because of their founding role and long history in the study of molecular biology. Microbial genomics also yields enormous and otherwise inaccessible informational gains compared to the relatively few easily observable phenotypes of microbes (at least compared to macroorganisms). This preponderance shows no signs of abating; the current Genomes Online Database (GOLD; http://www.genomesonline.org/cgi-bin/GOLD/bin/gold.cgi; accessed August 15, 2011) lists 8212 bacterial and 308 archaeal genome projects either completed or ongoing. These statistics do not include a myriad of sequenced viruses or projects not registered in GOLD.

For approximately 30 years following its original publication in 1977 [2], the Sanger dideoxy terminator method was the standard for DNA sequencing. Whereas this technology is now fully mature and capable of routinely yielding reads > 800 bp long, its reliance on capillary electrophoresis created a ceiling for the volume and speed of sequences that can be generated inexpensively by this approach [3]. So-called "next-" or "second-generation" sequencing approaches emerged in 2005 following the release of the 454 pyrosequencing platform (Roche), followed closely thereafter by the Illumina/Solexa (Illumina) and SOLiD (Applied Biosciences/Life Technologies) systems [3-6]. Second-generation sequencing platforms all produce reads shorter than those achieved by modern Sanger sequencing, although new PacBio and Roche FLX Titanium XL+ technologies may offer alternatives. Regardless, short-read sequencing remains the most cost-effective on a per-base basis (L. A. Pannacchio, unpublished data), leading to the dominance of these technologies (especially Illumina) in terms of market share http://www.genomicslawreport.com/wp-content/uploads/2011/04/JP-Morgan-NGS-Report.pdf.

Next-generation sequence assembly is an inherently challenging computational problem, stemming from the relatively low amount of data contained within each sequencing read, the huge volumes of data produced, and platform-specific error frequencies and profiles [7] including difficulties with regions of high %GC bias [8] and homopolymeric regions leading to frame-shifts [9]. Simulations suggest that completely unbroken coverage of a bacterial genome is impossible using short read lengths [10,11], although to some extent this can be compensated for using (more expensive) paired-end approaches [12]. Improving a draft-quality genome to completion is typically costly and laborious due to its general requirement for targeted PCR and Sanger sequencing. Fragmented genomes should therefore be the expectation from modern genome sequencing projects, at least for the foreseeable future, given the current economic reality and the market dominance of short-read sequencing platforms.

Consequently, data occurring between genomic contig ends are omitted from draft-quality genomes. This affects comparative analyses using phylogenetic techniques; because the state of these missing characters cannot be otherwise estimated, their phylogenetic analysis is impossible. In contrast, analyses based on presence-absence of particular open reading frame (ORF) types, e.g., using BLAST [13], can be performed using ORF fragments. These estimations are primarily susceptible to two types of errors: false-negatives, where ORFs that should be present are not annotated; and false-positives, where multiple fragments actually belonging to the same ORF are annotated separately. In the present work, we attempt to understand the extent and consequences of the biases partial ORFs introduce into annotation analyses. Furthermore, we attempt to mitigate these biases by linking fragmented ORFs based on their relatedness to homologs in closely-related organisms. We acknowledge that ORF fragmentation can introduce other biases into comparative analyses, e.g., due to the increased difficulty of correct ORF modeling [14] or potentially, misannotation due to the lower informational content of ORF fragments relative to full-length sequences. However, these errors fall outside the scope this current work.

## Results

### Extent of predicted ORF fragments in publicly available draft genomes

**Figure 1:**
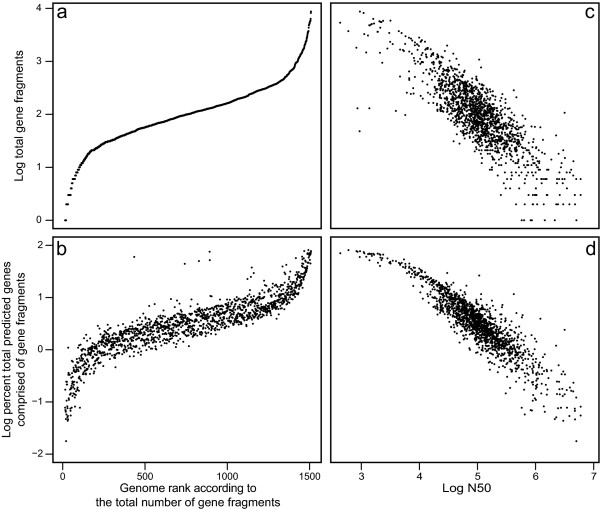
**Quality of draft genomes available in the NCBI database as of May 10, 2011**. The number of ORF fragments predicted by Prodigal and the percent of the total number of predicted ORFs (including ORF fragments) composed of ORF fragments, respectively, ordered by increasing number of ORF fragments (a and b) or plotted versus N50, the size of the contig for which 50% of the genome is contained in contigs of greater than or equal size (c and d). The number of ORF fragments, the percent of the total number of predicted ORFs composed of ORF fragments and N50 were logarithmically transformed to de-emphasize extreme values.

Whereas it is intuitive to expect ORF fragmentation in a draft genome due to the existence of multiple contigs, the extent of this problem is currently unknown. The majority of draft genomes in the NCBI database at the time of this study contained tens to hundreds of partial ORF fragments, although some fell both above and below this range (Figure 1a). The largest number of ORF fragments in a particular genome was 8,717 and the maximum percentage of the total number of predicted ORFs composed of partial sequences was 81.4% (Figure 1b). The increased numbers of ORF fragments is a result of decreased assembly quality, as indicated by the negative correlation of both the number of fragments and the percentage of the total number of predicted ORFs composed of partial sequences with N50 (Figures 1c and 1d). Note that, whereas N50 values from different genomes cannot be directly compared due to their dependence on genome size, the strong negative correlation with genome fragment abundance supports its use here as an estimate of genome quality. These distributions likely reflect the technological transition from Sanger sequencing towards 454-, Illumina- and SOLID-based methods.

### The effect of partial ORFs on annotation efficacy

As noted, genome fragmentation can affect ORF annotation either by erroneously omitting ORFs or by duplicating annotations for fragments originating from the same ORF. We examined the effect of fragmentation on ORF annotation in 25 *Streptomyces *genomes using three different annotation types, representing different annotation targets (e.g., Pfam primarily annotates domains whereas COG and KEGG focus more on the entire protein) and sensitivities (e.g., KAAS incorporates annotation heuristics [15] whereas COG and Pfam annotations were based solely on RPSBLAST). Genome assembly quality was approximated using the percentage of predicted ORFs composed of fragments and the average ORF length. Note that these metrics reflect genome quality oppositely: high quality genome assemblies have longer mean ORF lengths and lower percentages of partial ORFs.

Regardless of the genome quality metric used, ORF fragmentation caused significant over-annotation using KEGG and significant under-annotation using Pfam (Table 1). Some under-annotation was also observed using COG, although this was not as significant as that observed for Pfam (Table 1). Under-annotation using Pfam may reflect the increased omission of partial or entire domains due to ORF fragmentation. Over-annotation using KEGG suggests that this annotation type is especially sensitive. All trends remained, although with lowered statistical significance, when *Streptomyces *sp. PP-C42, the only genome in our dataset generated using solely Illumina sequencing [16], was excluded (Table 1). This may suggest that annotation difficulties are especially exacerbated by some applications of short-read genome sequencing technologies.

**Table 1:** Effect of genome quality on annotation efficacy

Uncorrected				
		Pfam^*a*^	COG^*a*^	KEGG^*a*^

Including PP-C42	% Partial ORFs fragments vs. % all ORFs annotated	*r *= -0.854 *P *< 0.001	*r *= -0.551 *P *= 0.012	*r *= 0.586 *P *= 0.007
	Mean ORF length vs. % all ORFs annotated	*r *= 0.785 *P *< 0.001	*r *= 0.526 *P *= 0.017	*r *= -0.403 *P *= 0.078
Excluding PP-C42	% Partial ORF fragments vs. % all ORFs annotated	*r *= -0.421 *P *= 0.073	*r *= -0.019 *P *= 0.939	*r *= 0.415 *P *= 0.078
	Mean ORF length vs. % all ORFs annotated	*r *= 0.406 *P *= 0.084	*r *= 0.157 *P *= 0.520	*r *= -0.016 *P *= 0.949

Corrected using matched partial ORF sets				

		Pfam	COG	KEGG

Including PP-C42	% Partial ORFs fragments vs. % all ORFs annotated	*r *= -0.861 *P *< 0.001	*r *= -0.595 *P *= 0.007	*r *= 0.469 *P *= 0.050
	Mean ORF length vs. % all ORFs annotated	*r *= 0.787 *P *< 0.001	*r *= 0.563 *P *= 0.012	*r *= -0.284 *P *= 0.253
Excluding PP-C42	% Partial ORF fragments vs. % all ORFs annotated	*r *= -0.338 *P *= 0.170	*r *= 0.027 *P *= 0.915	*r *= 0.350 *P *= 0.168
	Mean ORF length vs. % all ORFs annotated	*r *= 0.378 *P *= 0.122	*r *= 0.155 *P *= 0.538	*r *= 0.052 *P *= 0.842

^*a*^Pearson correlations between annotation frequency and genome quality, as represented by the percent of the predicted ORFs composed of partial sequences and mean ORF length. Complete genomes are excluded in all cases; including them has essentially no effect.

### Identifying functional categories enriched in partial ORFs

**Figure 2:**
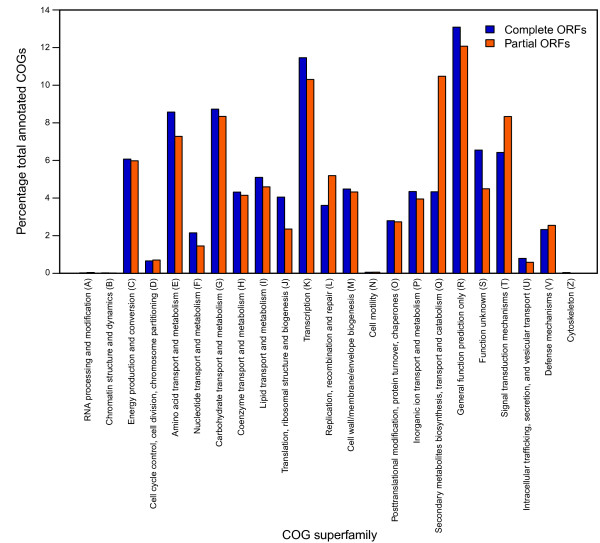
**Mean partial and complete ORFs for each COG superfamily**. The values shown are expressed as a percentage of the total number of partial or complete ORFs in the draft *Streptomyces *genomes examined. COG superfamily single-letter abbreviations are bracketed.

To further explore the effect of ORF fragmentation, we separately annotated the partial and complete ORFs according to their COG superfamilies in the draft genomes from our *Streptomyces *dataset (Figure 2). We aggregated the COG superfamily annotations for partial and complete ORFs in this analysis because the low number of partial fragments in some genomes became non-representative when converted to a percentage. Furthermore, the average percentage of ORFs belonging to some COG superfamilies for these genomes exhibited a strongly bimodal distribution (i.e., some genomes differed greatly between partial and complete ORFs, whereas others did not), somewhat undermining statistical inferences based on the mean. Despite these limitations, most COG superfamilies were represented by complete and partial ORFs approximately equally (Figure 2). This suggests that ORF fragmentation is a largely stochastic process driven by local, sequence-specific characteristics. However, three COG superfamilies were substantially enriched in partial ORF fragments: "replication, recombination and repair", "signal transduction mechanisms" and "secondary metabolites biosynthesis, transport and catabolism" (Figure 2). Inspection of the underlying data revealed that these enrichments were almost entirely due to three COG families: COG1020--non-ribosomal peptide synthetase (NRPS) modules and related proteins, on average 0.26% of complete ORFs but 2.25% of partial ORFs; COG3321--polyketide synthase (PKS) modules and related proteins, on average 0.20% of complete ORFs but 6.70% of partial ORFs; and COG0515--serine/threonine protein kinase, on average 0.41% of complete ORFs but 2.01% of partial ORFs. COG1020 and COG3321 both belong to the "secondary metabolites biosynthesis, transport and catabolism" superfamily, and COG0515 belongs to both the "replication, recombination and repair" and "signal transduction mechanisms" superfamilies. Fragmentation of PKSs, NRPSs and serine/threonine kinases are likely due to their multi-modular structures which are composed of homologous modules [17,18]. The repetitive nature of these enzymes, along with the lower per-base information content inherent to the GC-bias characteristic of actinomycetes, is likely to complicate genome-based drug-discovery efforts based on short-read sequencing platforms.

### Linkage of partial ORFs using homologs in related genomes

Whereas missing data in fragmented genomes cannot be compensated for without improvements to the genome sequencing and assembly procedure itself, the same is not necessarily true for fragments leading to falsely inflated annotation estimates. We hypothesized that linkage between some fragmented ORFs could be inferred based on their common homology to complete ORFs in other genomes. After extensive parameterization to find the most sensitive and specific similarity and coverage thresholds for fragment assembly using our algorithm (Additional files 1 and 2), a substantial proportion of ORF fragments could be so linked, ranging from 9.3% for *S. clavuligerus *ATCC27064 #2 up to 46.2% for *S. roseosporus *NRRL11379 (Table 2). The parameters used yielded a low overall false positive rate for the entire dataset (< 2%) as determined by the congruency of the linked ORF fragment sets with scaffold information (Additional files 1 and 2).

**Table 2:** Efficacy of partial ORF linkage^*a*^

Strain	True matching fragments linked (%)	**False matching fragments linked (%)**^***b***^
*S. albus *J1074	127 (22.1)	2 (0.4)
*S. clavuligerus *ATCC27064 (#1)	58 (31.2)	0 (0)
*S. clavuligerus *ATCC27064 (#2)	40 (9.3)	0 (0)
*S. clavuligerus *ATCC27064 (#3)	12 (20.7)	2 (3.5)
*S. ghanaensis *ATCC14672	182 (28.04	0 (0)
*S. griseoflavus *Tü4000	326 (27.5)	2 (0.2)
*S. hygroscopicus *ATCC53653	220 (20.2)	1 (0.1)
*S. lividans *TK24	117 (27.9)	2 (0.5)
*S. pristinaespiralis *ATCC25486	207 (20.7)	0 (0)
*S. roseosporus *NRRL11379	182 (46.2)	7 (1.8)
*S. roseosporus *NRRL15998	192 (41.6)	8 (1.7)
*Streptomyces *sp. XylebKG-1	0 (0)	0 (0)
*Streptomyces *sp. C	146 (17.9)	9 (1.1)
*Streptomyces *sp. e14	158 (17.7)	2 (0.2)
*Streptomyces *sp. Mg1	46 (12.0)	0 (0)
*Streptomyces *sp. PP-C42	887 (10.6)	No scaffolds
*Streptomyces *sp. SPB74	145 (15.8)	12 (1.3)
*Streptomyces *sp. SPB78	109 (12.8)	6 (0.7)
*S. sviceus *ATCC29083	200 (28.1)	7 (1.0)
*S. viridochromogenes *DSM40736	102 (31.1)	8 (2.4)

^*a *^All *Streptomyces *genomes except *Streptomyces *sp. PP-C42 and each query genome were used to link partial ORFs in that query genome. The parameters used were: ≥ 20% identity to and ≥ 60% coverage between the query and reference sequence and ≥ 50% similarity between the identities of each partial fragment to the reference sequence.

^*b*^False positive linkages were identified from their incongruencies with the scaffold information

**Figure 3:**
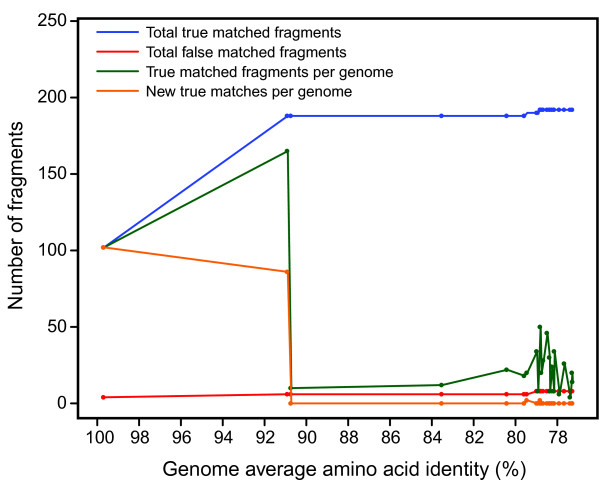
**The effect of genome relatedness on partial ORF linkage**. Cumulative and genome-specific linkage of ORF fragments are plotted according to increasing AAI divergence between *S. roseosporus *NRRL15998 and the other genomes in the *Streptomyces *test dataset. Reference genomes were ordered for analysis by their decreasing AAI to *S. roseosporus *NRRL15998.

**Figure 4:**
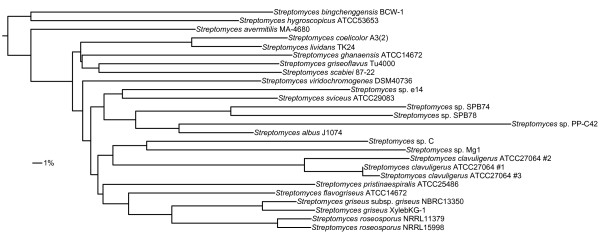
**Phylogenetic tree of the *Streptomyces *genomes used in this study**. This neighbor-joining phylogenetic tree is based on the average amino acid identity, calculated using each non-fragmented bidirectional best BLAST pair having ≥ 30% identities over ≥ 70% of both protein lengths. The tree was rooted based on a 16S rRNA gene tree constructed for all actinobacterial type strains which was consistent in all major respects to the tree presented here.

Inspection of the relationship between genetic relatedness and partial ORF linkage revealed insights into the varied rate of partial ORF linkage observed between genomes in our test dataset (Figure 3). In some cases, genome relatedness influenced partial ORF linkage, with genomes having average amino acid identities (AAIs) < 80% (the majority of the dataset; Figures 3 and 4) linking few sets of partial ORFs (Figure 3). However, many exceptions to this trend could also be found resulting and an overall lack of correlation between the AAI between two compared genomes and the number of linked fragments (R^2 ^= 0.006; *P = *0.061). Using the percentage of orthologous full-length proteins as the metric of genome similarity yielded equivalent results (data not shown). Even more important than genome relatedness *per se *was the degree to which related samples sampled the variable genome of the taxon of interest [19]. Both the influence and variability in this parameter are exhibited by *S. roseosporus *NRRL15998, for which more partial ORFs were linked using *Streptomyces *sp. XylebKG-1 as a reference (90.9% AAI, 165 sequences matched) compared with either *S. roseosporus *NRRL11379 (99.7% AAI, 102 sequence matched) or *S. griseus *subsp. *griseus *NBRC13350 (90.7% AAI, 10 sequences matched; Figure 3). (*Streptomyces *sp. XylebKG-1 and *S. griseus *subsp. *griseus *NBRC13350 are 98.4% related to each other by AAI; Figure 4.) Note that 95-96% AAI correlates to 70% DNA-DNA hybridization, i.e., bacterial species as currently defined [20]. In contrast, the three replicate *S. clavuligerus *ATCC27064 genomes (derivatives of the same strain sequenced by different institutes) could link relatively few of each other's partial ORFs despite the high relatedness of these genomes (Table 2). This may indicate that: (i) some genome fragments are inherently not linkable using homology e.g., due to the presence of multiple, closely related paralogs in a genome; (ii) some highly similar genome regions may fragment similarly, requiring slightly more divergent homologs for linkage; (iii) even extremely highly related genomes (such as the *S. clavuligerus *ATCC27064 replicates) can differ in some respect, e.g., plasmid content [21]. Regardless, these results suggest that this homology-based approach to fragment linkage might become more effective as more related genomes become available, as is expected due to the ever-decreasing cost of genome sequencing. However, selecting appropriate reference genomes remains somewhat empirical due to the current lack of a strong correlation between genome similarity and fragment linkage.

To determine whether our partial ORF linkage method could compensate for errors in KEGG over-annotation, we tested the correlation between the number of KEGG annotations after correction and both the mean ORF length and percentage of the total number of ORFs composed of fragments as described above (Table 1). All correlations between the proxies used to represent genome quality and the percentage of KEGG annotations decreased in significance or became statistically non-significant following fragment linkage. These results suggest that over-annotation can at least be partially compensated for by fragment linkage, especially as the variable genome for each species becomes better sampled. Similar correction of the COG and Pfam annotations increased the degree of under-annotation where this was previously statistically significant, albeit only slightly (Table 1).

## Discussion

Next-generation DNA genome sequencing has irrevocably moved microbiology (and much of biology, for that matter) towards extensive data volumes and the concomitant challenges of their analyses. These advances are not without attendant compromises, especially the economic trade-off between dataset breadth and quality which appears to be increasingly towards the former at the expense of the latter. Emerging technologies such as PacBio and 454 FLX Titanium XL+ offer enhanced read lengths, but these formats still cannot match the per-base costs of shorter-read sequencing platforms. Cheaper short read techniques therefore seem poised to dominate the sequencing market for the foreseeable future, especially given that human genome re-sequencing, the main economic driver for innovation in genome sequencing technologies, is focused on clinical personal genomics where short reads appear largely sufficient for reference assembly and SNP detection.

Given this technological reality, draft genomes are likely to dominate microbial genomic data for the immediate future. Here, we have attempted to address some of the limitations of this data type for comparative genomics analysis. We find that ORF fragmentation has the potential to dramatically cause false-negatives in some annotation types, especially those relying on relatively small stretches of sequence (e.g., Pfam domains; Table 1). These biases will be difficult to address without improvements in genome quality. Reciprocally, we also find that ORF fragmentation can cause false-positive annotations of multiple ORF fragments belonging to the same complete ORF where sufficiently sensitive detection methods exist (e.g., KEGG; Table 1). However, we also suggest that these problems can likely be at least partially mitigated by linkage of protein fragments based on their complete homologs in related organisms (Table 1, Figure 3). The impact of a well-sampled variable genome on ORF fragment linkage is especially intriguing (Figure 3), and suggests that sampling multiple related strains will not only more accurately represent biological diversity but will also improve the data quality of all these strains by unmasking otherwise obscured homologies among ORF fragments. This is likely to be particularly important for *Streptomyces *and other similar organisms, for which the most biotechnologically interesting genes involved in secondary metabolism are both variable between strains [22] and highly fragmented (Figure 2), and assembly is particularly challenged by low per-read information content due to %GC bias.

Although our analyses indicate the potential for biases, they in no way diminish the value of draft bacterial genome sequencing. Indeed, as described above, taking advantage of low cost-sequencing is essential to begin generating sufficient representation of the vast microbial diversity present in nature. With this increased sampling we can begin to differentiate between neutral and adaptive (or at least conserved) genome contents. However, we must also be aware of the limitations of these data types. For example, if > 80% of the predicted ORFs in a given bacterial genome are likely to be fragmented, analyses that can accommodate ORF fragmentation, even if imperfectly, will be crucial. Extensively fragmented draft genomic data will be insufficient for some applications, even with downstream *in silico *correction. This will necessitate proper resource allocation to ensure data usability for a given experimental objective. Furthermore, data analysis requirements scale even faster than data accumulation, e.g., all-versus-all comparisons of *n *sequences most simply require *n^2 ^*comparisons. It may therefore be necessary to use judicious subsets of a global dataset when attempting fragment linkage. In the end, there is no substitute for well designed, tractable research; draft sequencing, for all its promise and pitfalls, is no different.

## Conclusions

Draft genome quality will be the dominant form of microbial genomic data produced for the immediate and foreseeable future. This approach produces highly fragmented genomes, which concomitantly translates into abundant predicted ORF fragments. These fragmented ORFs lessen annotation efficacy, especially of repeating, multi-modular proteins. However, this problem can be somewhat ameliorated by increased sampling of a species' variable genome. Therefore, despite their great utility in many applications, there exist disadvantages to draft genome sequencing that need to be addressed by appropriate experimental design and deployment of sequencing resources to effectively answer the study questions posed.

## Methods

### Dataset construction

The contigs comprising all 1,510 partial genome sequences were obtained from the NCBI FTP folder "genomes/Bacteria_DRAFT" on May 10, 2011. Genes and their corresponding proteins were annotated using Prodigal v2.00 [23] according to default parameters, and the numbers and fragmentation of predicted proteins, the N50 and the %GC for each genome enumerated using a custom Perl script. (N50 is the contig size for which 50% of the genome sequence exists in contigs of at least that size. We define %GC bias as deviation from 50% (C+G)/(A+C+G+T), as would be expected if all possible nucleotide substitutions were equally probable.) ORF fragments were automatically annotated by Prodigal as ORFs that were bounded by a contig end. The list of *Streptomyces *genomes used for more detailed analysis is given in Table 3. These genomes were selected because they vary considerably in quality, from complete genomes to one generated exclusively using Illumina sequencing. They also range considerably in their phylogenetic relatedness (Figure 4), including three separate sequencing projects for the *S. clavuligerus *ATCC27064 using derivatives of the same strain. *Streptomyces *genomes are also expected to be especially challenging to assemble using short-read techniques due to their substantial %GC bias [22]. All of the selected genomes (with the exception of *Streptomyces *sp. PP-C42) had accompanying scaffold information, which allowed estimation of the false positive rate of our partial ORF linkage procedure (see below). Annotated protein sequences were downloaded from NCBI for those strains having complete genomes; otherwise, proteins were predicted from nucleotide contigs using Prodigal v2.00 [23] as described above.

**Table 3:** Characteristics of the *Streptomyces *genomes used in this study

Strain	Contigs/ Scaffolds	Genome Size (bp)	N50 (bp)	**Complete/Partial ORFs (% Partial)**^***a***^	Ref.; NCBI project accession number
*S. albus *J1074	501/2	6,619,469	23,675	5442/575 (9.6)	The Broad Institute, unpublished; PRJNA37045
*S. avermitilis *MA-4680	Complete	9,119,895	-	7676/0 (0)	[25,26]; PRJNA47909
*S. bingchenggensis *BCW-1	Complete	11,936,683	-	10035/0 (0)	[27]; PRJNA35119
*S. clavuligerus *ATCC27064 (#1)	279/2	8,528,397	50,310	6893/186 (2.6)	[28]; PRJNA42475
*S. clavuligerus *ATCC27064 (#2)	597/158	6,729,086	16,887	5692/430 (7.0)	The Broad Institute, unpublished; PRJNA28551
*S. clavuligerus *ATCC27064 (#3)	89/5	9,134,976	203,387	7567/58 (0.8)	[21]; PRJNA19249
*S. coelicolor *A3(2)	Complete	9,054,847	-	8153/0 (0)	[29]; PRJNA35153
*S. flavogriseus *ATCC33331	Complete	7,656,104	-	6763/0 (0)	The Joint Genome Institute, unpublished; PRJNA37207
*S. ghanaensis *ATCC14672	616/3	8,223,278	24,170	7166/649 (8.3)	The Broad Institute, unpublished; PRJNA37041
*S. griseoflavus *Tu4000	927/1	7,364,052	14,024	6076/1187 (16.3)	The Broad Institute, unpublished; PRJNA37185
*S. griseus *subsp. *griseus *NBRC13350	Complete	8,545,929	-	7136/0 (0)	[30]; PRJNA36231
*S. griseus *XylebKG-1	4/2	8,731,583	603,0272	7374/5 (0.1)	[31]; PRJNA38545
*S. hygroscopicus *ATCC53653	783/2	10,466,286	27,794	8647/1092 (11.2)	The Broad Institute, unpublished; PRJNA37181
*S. lividans *TK24	333/1	8,190,887	49,556	7183/419 (5.5)	The Broad Institute, unpublished; PRJNA37179
*S. pristinaespiralis *ATCC25486	844/1	7,633,609	17,558	6283/1000 (13.7)	The Broad Institute, unpublished; PRJNA36845
*S. roseosporus *NRRL11379	280/2	7,763,119	57,471	6737/394 (5.5)	The Broad Institute, unpublished; PRJNA65085
*S. roseosporus *NRRL15998	371/1	7,560,086	38,584	6493/462 (6.6)	The Broad Institute, unpublished; PRJNA55545
*S. scabiei *87-22	Complete	10,148,695	-	8746/0 (0)	Wellcome Trust Sanger Institute, unpublished; PRJNA35395
*Streptomyces *sp. C	652/4	7,916,041	25,608	6906/815 (10.6)	The Broad Institute, unpublished; PRJNA37177
*Streptomyces *sp. e14	716/13	7,146,196	21,378	5780/893 (13.4)	The Broad Institute, unpublished; PRJNA38087
*Streptomyces *sp. Mg1	466/127	7,105,723	25,079	6344/384 (5.7)	The Broad Institute, unpublished; PRJNA36841
*Streptomyces *sp. PP-C42	7074/-	6,467,850	1,414	2503/8392 (77.0)	[16]; PRJNA63125
*Streptomyces *sp. SPB74	845/2	6,505,970	13,132	5401/917 (14.5)	The Broad Institute, unpublished; PRJNA36843
*Streptomyces *sp. SPB78	694/4	6,897,976	20,678	5723/853 (13.0)	The Broad Institute, unpublished; PRJNA37173
*S. sviceus *ATCC29083	552/1	9,055,790	33,523	7921/711 (8.2)	The Broad Institute, unpublished; PRJNA36847
*S. viridochromogenes *DSM40736	226/1	10,988,130	109,490	7473/328 (4.2)	The Broad Institute, unpublished; PRJNA37183

^*a *^Predicted proteins for complete genomes were downloaded directly from the NCBI database; all others were predicted using Prodigal.

### Gene annotation

The COG v1.0 and Pfam v25.0 databases were downloaded from the NCBI FTP site and queried by RPSBLAST with an expectation cut off of 1 × 10^-5 ^using the predicted proteins in the *Streptomyces *dataset. The top COG and all Pfam hits for each query sequence were recovered for analysis. A Pfam annotation conducted using an expectation cut off of 1 × 10^-3 ^yielded similar but slightly stronger statistical correlations compared with those shown in Table 1 (data not shown). KEGG classifications for the predicted proteins in the *Streptomyces *dataset were annotated using the KAAS web server using the single direction best BLAST method [15]. Statistical analyses were all conduced using R v2.13.1.

### Partial ORF linkage

Our partial ORF linkage algorithm was implemented as a Perl script, freely downloadable from the Currie lab website http://currielab.wisc.edu/. Note that this current implementation was designed to use pair-wise BLAST searches of entire proteomes due to the additional utility of these files for calculating AAIs. Other configurations (e.g., BLASTing only partial sequences) may be more computationally efficient depending on particular experimental needs. Pair-wise comparisons of each predicted proteome were conducted using BLASTp [13] with the following parameters: -a 8 -b 20 -v 20 -e 1e-05 -F F. AAIs were calculated according to Konstantinidis and Tiedje [20]. Pair-wise AAIs were averaged for each genome to construct a double-sided distance matrix, from which a neighbor-joining tree was constructed using NEIGHBOR in the PHYLIP package v3.69 [24].

**Figure 5:**
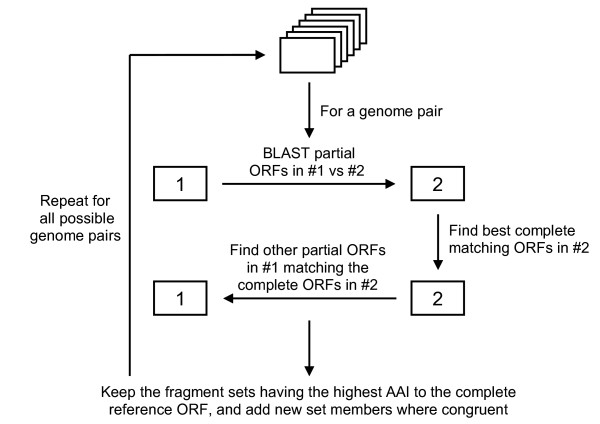
**Flow diagram of the partial ORF linkage approach used in this work**.

A flow diagram of our approach is presented in Figure 5. Essentially, complete homologs in genome #2 were sought for protein fragments predicted in genome #1, which were then used to detect other protein fragments predicted in genome #1 likely to comprise a matching set with the first predicted protein fragment. The stringency of this matching process was constrained by user specified thresholds for: (i) the minimum percent identity required to define homologs between genomes #1 and #2; (ii) the minimum percent overlap required between the homologs in genomes #1 and #2; (iii) the minimum difference in the percent identities of a set protein fragments to a complete reference homolog required to consider multiple predicted protein fragments in genome #1 as belonging to the same predicted protein fragment set; and (iv) the number of overlapping amino acids allowed between predicted protein fragments in a set. Searches were also conducted reciprocally using homologs in genome #1 to find matching sets of predicted partial proteins in genome #2. The fragment-matching process was further constrained by allowing only two predicted partial proteins truncated at either their 5'- or 3'-ends in the same predicted protein fragment set; no limit was set for predicted partial proteins truncated at both their 5'- and 3'-ends. Note that this step is especially sensitive to errors in predicting whether a protein is truncated at one or both ends, which were often encountered in our test dataset, but was included to be more conservative. Finally, this same matching process was repeated using multiple predicted proteomes, with matched sets being rejected and replaced if alternative sets are found in another predicted proteome that satisfy the above criteria but have a higher mean AAI between the matched protein fragments and their complete protein homologs in another proteome. This step assumes that homologs with higher AAIs are more likely to be orthologous than more divergent ones and are therefore more reliable as scaffolds for matching partial protein fragments. We note that orthology is not an explicit requirement for fragment recruitment; a reference paralogous protein might the closest homolog to a query when the ortholog in that reference genome has been lost. Whereas we used translated protein sequences for fragment linkage to better compensate for the genetic divergence between the genomes that we analyzed, nucleotide sequences could be similarly analyzed if several closely-related genomes are available.

## Authors' contributions

JLK conceived of the study, participated in its design, performed the analyses, analyzed the results and drafted the manuscript. CRC participated in this study's design, helped analyze data and draft the manuscript. Both authors have read and approved the final manuscript.

## Supplementary Material

Additional file 1 **Parameterization of the fragment linkage algorithm by varying the maximum percentage identities, sequence overlap and minimum difference in the percent identities of a set protein fragments to a complete reference homolog**. False and true positive linkage results were defined based on their concordance with the adjacent location of each fragment pair on the same contig. Each analysis was conducted on the entire *Streptomyces *test dataset except for *Streptomyces *sp. PP-C42, for which there was not available scaffold information. Click here for file

Additional file 2 **Parameterization of the fragment linkage algorithm by varying the maximum percentage of identities and sequence overlap while holding the minimum difference in the percent identities of a set protein fragments to a complete reference homolog constant at 40%**. The height of the surface represents the number of fragments matched using each parameter combination, and the color represents the percentage of true positives recovered using those parameters by reference to the available scaffold information. Click here for file
