# Supplementary material for: Gene fragmentation in bacterial draft genomes: extent, consequences and mitigation
Source: BMC Genomics. 2012 Jan 10;13:14. doi: 10.1186/1471-2164-13-14 (PMC3322347; doi:10.1186/1471-2164-13-14)
Supplement: Additional file 2 — Parameterization of the fragment linkage algorithm by varying the maximum percentage of identities and sequence overlap while holding the minimum difference in the percent identities of a set protein fragments to a complete reference homolog constant at 40%. The height of the surface represents the number of fragments matched using each parameter combination, and the color represents the percentage of true positives recovered using those parameters by reference to the available scaffold information. [file 1471-2164-13-14-S2.PDF]

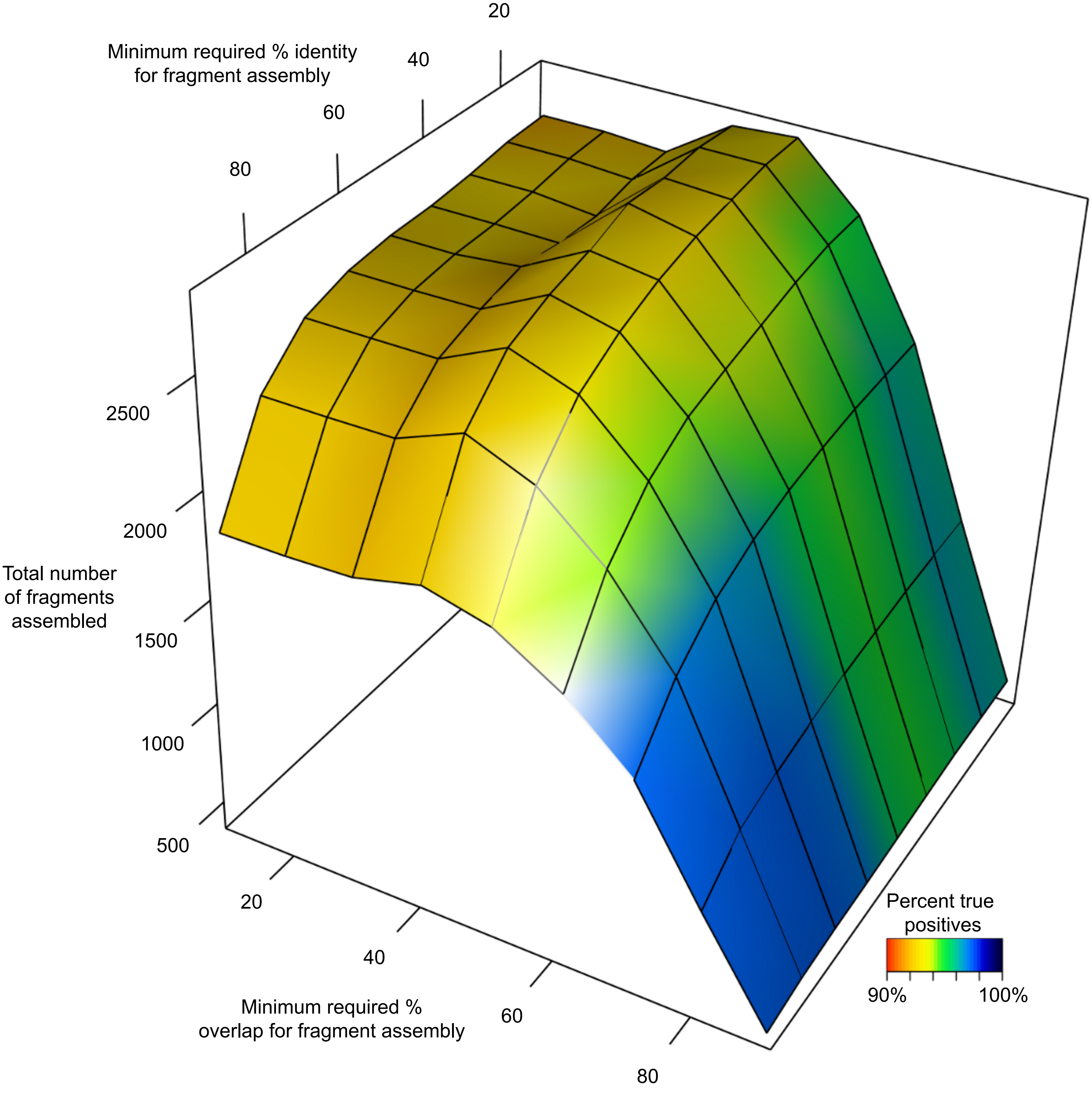

Additional file 2: Contig_assembly_parameters.pdf
Parameterization of the fragment linkage algorithm by varying the maximum percentage of identities and sequence overlap while holding the minimum difference in the percent identities of a set protein fragments to a complete reference homolog constant at 40%. The height of the surface represents the number of fragments matched using each parameter combination, and the color represents the percentage of true positives recovered using those parameters by reference to the available scaffold information.
